# Thymic Stromal Lymphopoietin and Cancer: Th2-Dependent and -Independent Mechanisms

**DOI:** 10.3389/fimmu.2020.02088

**Published:** 2020-09-16

**Authors:** Maria Pia Protti, Lucia De Monte

**Affiliations:** ^1^Tumor Immunology Unit, Istituto di Ricerca a Carattere Scientifico (IRCCS), San Raffaele Scientific Institute, Milan, Italy; ^2^Division of Immunology, Transplantation and Infectious Diseases, San Raffaele Scientific Institute, Milan, Italy

**Keywords:** TSLP, tumor cells, cancer associated fibroblasts, dendritic cells, Th2 inflammation, CD4^+^ Th2, TSLPR, IL-1

## Abstract

The thymic stromal lymphopoietin (TSLP) is an IL-7-like cytokine originally cloned from a murine thymic stromal cell line, and subsequently a human homolog was identified using database search methods. Human TSLP is mostly expressed in epithelial cells, among which are keratinocytes as well as stromal cells such as fibroblasts and immune cells. Human TSLP was first described to activate myeloid dendritic cells, which prime naïve T helper cells to produce high concentrations of Th2 cytokines, thus representing a key cytokine in triggering dendritic cells-mediated allergic Th2 inflammation. TSLP and/or its receptor has been shown to be expressed in several tumor types, where TSLP expression is associated with functional activities that can be associated or not with the induction of a Th2-prone tumor microenvironment, i.e., Th2-dependent and Th2-independent mechanisms. These mechanisms involve tissue- and immune cell target-dependent tumor-promoting or tumor-suppressive functions in different or even the same tumor type. Here we report and discuss the Th2-dependent and Th2-independent roles of TSLP in cancer and possible therapeutic targeting.

## Introduction

The thymic stromal lymphopoietin (TSLP) is an IL-7-like cytokine originally cloned from a murine thymic stromal cell line (1), and a cDNA clone encoding human TSLP was then identified using database search methods (2, 3). A low affinity TSLP receptor (TSLPR) was isolated (4–7), most closely related to the common γ-chain (6). Subsequently, a functional high-affinity TSLPR complex was defined as a heterodimer formed by the TSLPR and the IL-7 receptor α-chain (5, 6). This receptor combination results in predominant STAT-5 activation and increased cell survival, proliferation, and differentiation to TSLP stimulation (2, 8–10).

TSLP is primarily expressed by epithelial cells at barrier surface, with the highest levels in skin, gut, and lung (11). Expression has been also described in smooth muscle cells and fibroblasts (12). Moreover, dendritic cells (DCs) (13), and possibly other immune cells such as mast cells, can produce TSLP (14). Analysis of the expression profile of TSLPR and IL-7 receptor α-chain subunits showed the highest co-expression of the two receptors in myeloid DCs (3). Several other immune cells from the innate (i.e., macrophages, monocytes, masts cells, neutrophils, eosinophils, NKT cells, and ILC2 cells) and adaptive (i.e., B cells, T cells, Th2 cells, CD8^+^ T cells, and regulatory T cells [Tregs]) immunity are a cellular target for TSLP, as well as other non-immune cells, such as platelets and sensory neurons (14, 15). TSLP expression is induced by proinflammatory stimuli, comprising IL-1 (16, 17).

TSLP had been initially implicated in allergic diseases, where it creates a predominant Th2 microenvironment, mostly through DC activation (i.e., upregulation of OX40L, CD80, and CD86) (18), by phosphorylation of several STAT proteins and NFκB (8, 10). More recently, a role for TSLP has been also reported in chronic inflammatory and autoimmune disorders and in cancer (14, 15). We refer readers interested in comprehensive synopses on the role of TSLP in several disease settings to those reviews. Here, we focus exclusively on the literature regarding TSLP expression and function in cancer with special emphasis on the association or not with Th2 inflammation.

## TSLP Isoforms in Cancer

Two TSLP isoforms have been identified in human bronchial epithelial cells (19) and are termed long- (i.e., the original one) and short-form TSLP. The short-form TSLP mRNA is constitutively expressed in bronchial and colonic epithelial cells, keratinocytes, and lung fibroblasts (19). Short-form TSLP is believed to exert homeostatic and anti-microbial activities (15, 20), and expression of one or the other or both isoforms in barrier surface diseases have been reported (20).

In cancer the expression of the two isoforms was evaluated in breast (21) and pancreatic (22) cancers. In breast cancer cells both isoforms were upregulated upon stimulation with IL-1β (21). In pancreatic cancer associated fibroblasts (CAFs), variable levels of short-form TSLP mRNA were expressed at the steady state that did not significantly increase upon activation, whereas long-form TSLP mRNA levels significantly increased after activation with proinflammatory cytokines (22), suggesting that the inducible form of TSLP was primarily the long one.

## TSLP in Cancer: Historical Perspective

The first identification of a role for TSLP in cancer was in pancreatic (23) and breast cancers (24, 25), in which TSLP, secreted by either CAFs or tumor cells, respectively, was found to exert tumor-promoting functions through the establishment of predominant Th2-type inflammation in the tumor microenvironment. Previous studies from the same authors reported the presence of carcinoembryonic antigen-specific Th2 cells in the blood of pancreatic cancer patients undergoing surgery that correlated with the presence of predominant GATA-3 positive lymphoid cells in the tumor stroma (26), and of inflammatory IL-13 secreting Th2 cells in primary breast cancer that contributed to accelerate tumor development in a humanized mouse model (27). In addition, in the 4T1 mouse model of breast cancer, an allergic response in the lung was required to favor metastasis formation (28). These data prompted the authors to look for mechanisms leading to Th2 inflammation in these tumors, and they hypothesized that, due to its function in Th2 allergic responses, TSLP could be a relevant candidate to investigate.

Following the first reports in pancreatic and breast cancer, several studies also in other tumors found either pro-tumor or anti-tumor activity of TSLP, and through Th2-dependent as well as Th2-independent mechanisms. This distinction is mostly based on the association between TSLP expression and the development of predominant Th2 inflammation in the tumor or direct TSLP signaling on TSLPR expressing tumor cells. These studies are summarized in Table 1.

**Table 1:** TSLP expression and pro-tumor or anti-tumor function in human and mouse cancers.

**Tumor type**	**TSLP expression**	**TSLP function**	**Human/ Mousemodels**	**Th2-dependent mechanisms**	**Th2-independent mechanisms**	**Clinical correlates**	**References**
Pancreatic cancer	CAFs	Pro-tumor	Human	CAF-derived TSLP activates myeloid DCs with Th2 polarizing capability (IL-13 producing CD4 T cells). CD11c^+^TSLPR^+^ cells are present in the tumor and tumor draining LNs		GATA-3^+^/T-bet^+^ cells ratio is an independent predictive factor of survival after surgery in pancreatic cancer patients	(23)
Pancreatic cancer	Skin keratinocytes, systemic	Anti-tumor	KPC cells (29) subcutaneously implanted in K14-TSLP^tg^ mice	GATA-3 cells in the tumor of K14-TSLP^tg^ are significantly increased compared to WT mice		Tumors in K14-TSLP^tg^ grow less than in WT mice	(30)
Breast cancer	Tumor cells	Pro-tumor	Human and humanized NOD/SCID/β2m KO mice subcutaneously implanted with human breast cancer cells	Tumor cell-derived TSLP activates myeloid DCs with Th2 polarizing capability (IL-13 and TNFα producing CD4 T cells). CD11c^+^OX40L^+^ are present in the tumor		Anti-OX40L and anti-TSLP antibodies significantly prolong survival in humanized immune-deficient mice	(25)
Breast cancer	4T1 tumor cells	Pro-tumor	4T1 cells subcutaneously implanted in TSLPR KO mice	TSLP-TSLPR signaling in Th2 cells		4T1 cells grow significantly less in TSLPR KO mice and give less metastases in the lung	(24)
Breast cancer	4T1 tumor cells	Pro-tumor	4T1 cells subcutaneously implanted in TSLPR KO mice	Immune response is shifted toward Th1 in TSLPR KO mice		4T1 cells grow significantly less in TSLPR KO mice and give less metastases in the lung but more in the brain	(31)
Breast cancer	Skin, systemic	Anti-tumor	K14-TSLP^tg^PYMt^tg^ mice PYMt^tg^ cells implanted in TSLPR KO	GATA-3 cells in the tumor of K14-TSLP^tg^PYMt^tg^ are significantly increased compared to single transgenic PYMt^tg^ mice		Tumor lesion numbers are significantly lower in double transgenic mice	(30)
Breast cancer	Myeloid cells	Pro-tumor	Orthotopic implant of TSLP- and TSLPR-deficient 4T1 cells MMTV-PyMT mice		Tumor-derived IL-1α induces TSLP expression in myeloid cells that in turn activated anti-apoptotic pathways in TSLPR^+^ tumor cells TSLP expression in lung is necessary for metastases	TSLP deficient mice implanted with 4T1 cells have smaller primary tumors and fewer lung metastases than WT mice Lung metastases are reduced by anti-TSLP antibody	(32)
Breast cancer	4T1 and KCMH-1 cells	Pro-tumor	4T1 orthotopic implantation in syngeneic mice		Tumor-derived TSLP induces the expression of tissue remodeling and angiogenic genes in alveolar macrophages	Reduced lung metastases in TSLP-KO bearing mice	(33)
Breast cancer	4T1 cells	Pro-tumor	4T1 orthotopic implantation in syngeneic mice TSLPR KO mice		TSLP promotes pre-B cell emigration from the bone marrow, and their survival/expansion in the periphery. Tumor cells favor conversion of pre-B cells into regulatory B cells that affect antitumor immunity and favor lung metastases	Reduced lung metastases in TSLP-KO bearing mice	(34)
Lung cancer	Tumor cells	Pro-tumor	Human		TSLP-conditioned DCs induce Tregs TSLP expression in the tumor correlates with the number of FoxP3^+^ Tregs	TSLP expression correlates with pathologic type, stage, tumor size, and LN metastases	(35)
Cervical cancer	Tumor cells	Pro-tumor	Human		Tumor-derived TSLP induces recruitment and proliferation of eosinophils that in turn promote tumor cell proliferation and inhibit apoptosis		(36)
Cervical cancer	Tumor cells	Pro-tumor	Human		TSLP promotes angiogenesis through eosinophil-derived factors		(37)
Cervical cancer	Tumor cells	Pro-tumor	Human		TSLP promotes tumor cell proliferation and invasion		(38)
Skin cancer	Keratinocytes	Anti-tumor	Mice KO in Notch signaling CD1 mice treated with DBA and TPA	Tumors in KO compared to WT mice are infiltrated by an higher percentage of Th2		Blocking TSLP signaling induces skin tumorigenesis	(39)
Skin cancer	Keratinocytes	Anti-tumor	Notch and/or β-catenin mutant mice crossed with TSLPR KO mice Mice constitutively expressing β-catenin and TSLPR KO		TSLP-TSPR signaling increased CD8 T cell fitness and reduced CD11b+Gr1+ cells	TSLP-TLSPR signaling protects against tumor formation	(40)
Skin cancer	Keratinocytes	Anti-tumor	Barrier protein deficient mice (EPI-/-) treated with DMBA and TPA		TSLP and NKG2D restrained skin carcinogenesis		(41)
Cutaneous T-cell lymphoma	Keratinocytes	Pro-tumor	Human and EL-4 and MBL-2 cell model	TSLP induces IL-4 and IL-13 expression by tumor cells through STAT5 activation	TSLP signaling induces proliferation of TSLPR+ tumor cells	Anti-TSLP antibody in mouse models reduces tumor formation	(42)
Colorectal cancer	Tumor cells	Anti-tumor	Human and xenograft model (subcutaneous injection of human tumor cells in nude mice)		TSLP-TSLPR signaling induces apoptosis	TSLP administration in mouse models inhibits colon tumor growth	(43)
Gastric cancer	Tumor cells	Pro-tumor	Human			TSLP overexpression correlates with LN metastases	(44)
Gastric cancer	Tumor cells	Pro-tumor	Human	Previous report (45) showed that infection by H. pylori induces release of TSLP from gastric cells that in turn trigger a Th2 response through DC activation		Prognosis in patients with TSLP^+^ tumor is worse than in patients with TSLP^−^ tumors TSLP serum levels are independent prognostic indicators	(46)
Ovarian carcinoma	Tumor cells	Pro-tumor	Human			TSLP is an independent predictive factor of reduced survival	(47)
Oropharyngeal squamous cell carcinoma	Tumor cells	Pro-tumor	Human	High IFNγ, and low IL-4, TSLP, and TGF-β correlates with increased survival		Low TSLP expression is a good prognostic factor	(48)
B cell precursor acute lymphoblastic leukemia	Not reported	Pro-tumor			TSLP-TSLPR signaling induces tumor cell proliferation and signal transduction		(49)

## Th2-Dependent Mechanisms of TSLP in Cancer

Chronic inflammation is associated with tumor development and progression (50, 51). While Th1-dependent acute inflammation has been associated with tumor rejection, Th2-dependent chronic inflammation is believed to enable tumor growth (52, 53). As mentioned above, TSLP promotes predominant Th2-type inflammation in different tumors and mediates pro-tumor but also anti-tumor functions (Table 1). In order to exert its Th2 polarizing effects, TSLP can either indirectly act through myeloid DC conditioning that supports Th2 cell priming/differentiation from naïve CD4 T cells (18) or directly bind to CD4^+^ T cells, which upregulate the TSLPR upon activation (54, 55), with higher expression on Th2 compared with Th1 and Th17 cells (9), suggesting that direct TSLP-TSLPR signaling occurs in antigen-specific memory T cells.

Th2-dependent mechanisms of TSLP in cancer have been reported in pancreatic, breast, skin, gastric, and oropharyngeal cancers, with pro- and anti-tumor effects, as detailed below.

### Pancreatic Cancer

**Figure 1:**
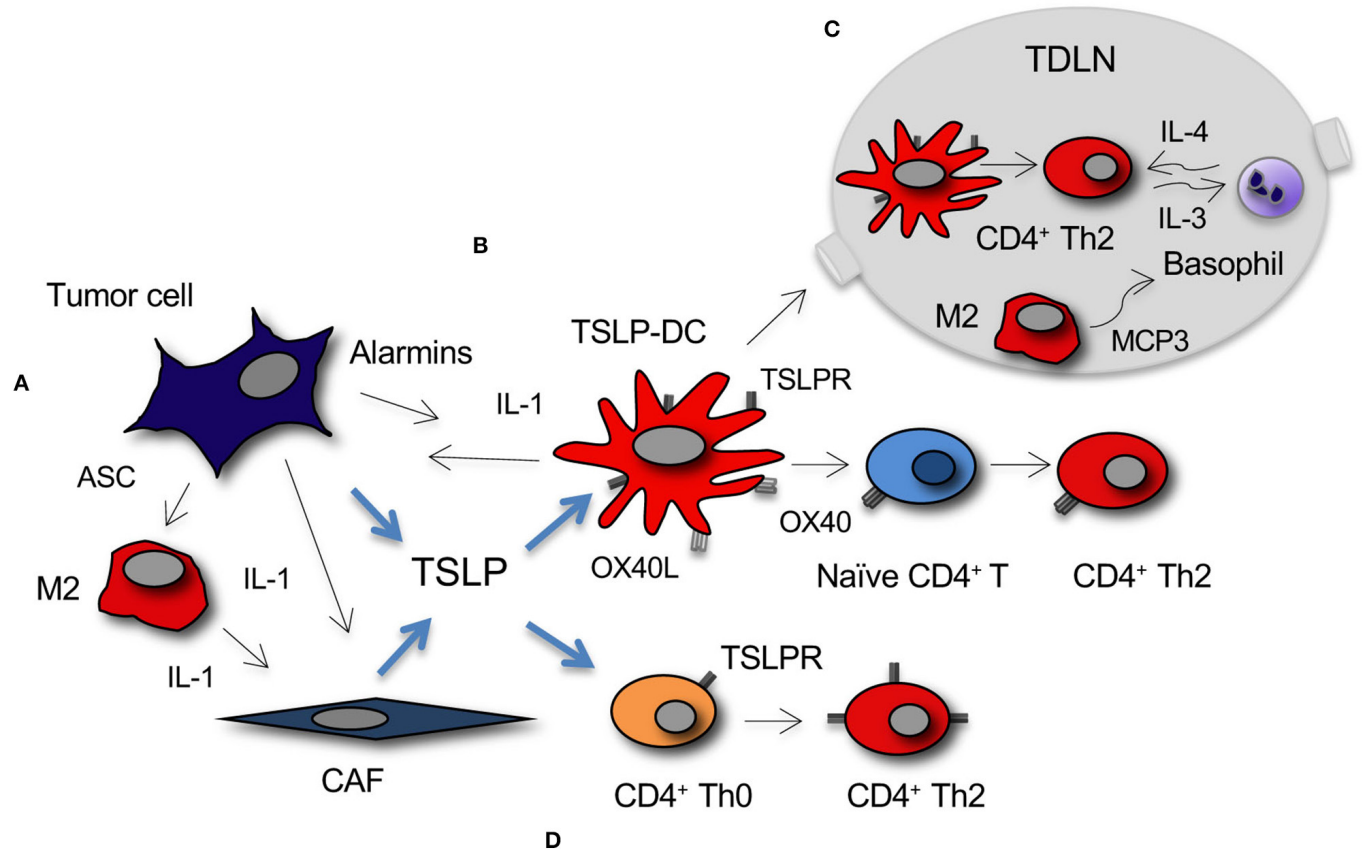
Development of TSLP-dependent protumor Th2 inflammation in cancer. **(A)** TSLP secretion by tumor cells and CAFs is primarily induced by tumor-derived IL-1. IL-1 is released directly from tumor cells or myeloid DCs and M2 macrophages within the stroma under the influence of tumor cell-derived alarmins, among which the inflammasome adaptor ASC. **(B)** Myeloid DC conditioned by TSLP (TSLP-DC) prime and polarize naïve CD4^+^ T cells toward a Th2 phenotype through TSLP-TSLPR and OX40-OX40L signaling. **(C)** TSLP-DC migrate to TDLNs where they prime CD4^+^ Th2 cells that secrete IL-3. M2 macrophages present in TDLNs secrete MCP3 that recruit basophils, which after activation by Th2-derived IL-3, secrete the IL-4 required for GATA-3 stabilization in CD4^+^ Th2 cells. **(D)** TSLP directly induces CD4^+^ T cell polarization toward Th2 and/or expansion of Th2 effectors through TSLP-TSLPR signaling.

A tumor-promoting function for TSLP was demonstrated in pancreatic cancer, where predominant Th2 (GATA-3^+^) over Th1 (T-bet^+^) cells within the lymphoid infiltrate in the tumor stroma was associated with reduced survival in pancreatic cancer patients, thus implying an active role for Th2 immunity in tumor progression (23). TSLP expression in the tumor was significantly higher than in the surrounding tissue, and, as reported above, it was supported by CAFs activated by tumor-derived cytokines. *In vitro* studies demonstrated that DCs activated with the supernatant of activated CAFs induced TSLP-dependent Th2 cell polarization of naïve CD4^+^ T cells (Figure 1B). Importantly, *in vivo* TSLPR expressing DCs were present in the tumor stroma and in tumor-draining but not in non-draining lymph nodes (LNs). The following studies identified a complex crosstalk in the tumor microenvironment and tumor-draining LNs (TDLNs) relevant to the establishment of TSLP-dependent Th2-type inflammation in pancreatic cancer. The authors reported that tumor-derived IL-1, released by tumor cells and inflammasome adaptor ASC-activated M2 cells, is crucial for TSLP secretion by CAFs (22) (Figure 1A), and that IL-4 derived by basophils, recruited into TDLNs by alternatively activated M2 macrophages, stabilizes the Th2 polarization (56) (Figure 1C), thus adding further complexity to the crosstalk within the tumor microenvironment that leads to predominant Th2 inflammation in pancreatic cancer (57). M2 macrophages and CD4^+^ Th2 present in the tumor microenvironment possibly mediate tumor progression by favoring invasion and metastasis formation, as it has been shown in a breast cancer model (58).

In contrast with the data reported in the human disease, tumor growth was reduced in a transplantable mouse model of pancreatic cancer, where transgenic mice overexpressing TSLP in the skin (K14-TSLPtg) were subcutaneously injected with syngenic pancreatic cancer cells, compared to the wild-type (WT) controls (30). Tumors from these transgenic mice had increased numbers of infiltrating CD4^+^ Th2 cells compared to WT mice, suggesting that in this model TSLP and Th2 cells exerted tumor-suppressive function in the context of a systemic Th2-polarized environment.

At difference with the transplantable K14-TSLPtg mouse model reported above (30), very recently DePinho and collaborators (59), using a transgenic mouse model of pancreatic cancer carrying an inducible oncogenic KRAS mutation, demonstrated a tumor-promoting function for Th2 cytokines from the tumor microenvironment, thus recapitulating the human disease. In this model, activation of cancer cells carrying the mutated KRAS by IL-4 and IL-13, which were secreted by the Th2 cells present in the tumor microenvironment, triggered the JAK1-STAT6-MYC pathway that in turn activated glycolysis crucial for tumor progression.

### Breast Cancer

Concomitantly and similarly to human pancreatic cancer, a tumor-promoting role for TSLP was demonstrated in breast cancer (25). The authors showed that human breast cancer cells directly produce TSLP, and that tumor cell derived-TSLP induces *in vitro* OX40L expression on DCs (25) (Figure 1B). OX40L-expressing DCs were found in primary breast tumor infiltrates and *in vitro* they drove the development of inflammatory Th2 cells (i.e., producing IL-13 and TNF-α). Importantly, in a xenograft mouse model, anti-TSLP or anti-OX40L neutralizing antibodies inhibited breast tumor growth and IL-13 production. Studies from the same group (21) showed that, similarly to pancreatic cancer (22), IL-1β, which was released by myeloid DCs under the influence of tumor-derived factors (i.e., alarmins), was key for TSLP secretion by breast cancer cells (Figure 1A).

A role for TSLP in breast cancer progression and metastasis to the lungs was also reported in Olkhanud et al. (24). In the 4T1 orthotopic murine model TSLP was produced by cancer cells that directly acted on TSLPR-expressing CD4^+^ T cells to induce their Th2 differentiation (Figure 1D). TSLP was also expressed by human breast metastases in the lung, and in the murine model the metastatic potential of different 4T1 cell clones was associated with their ability to secrete TSLP. *In vivo* TSLP functional inactivation either by silencing or by using TSLPR KO mice demonstrated the role of tumor-derived TSLP in inducing a metastases prone environment in the lungs. This was due to secretion by CD4^+^ T cells of pro-tumor Th2 cytokines (i.e., mainly IL-10 and IL-13), possibly with activation of CD4^+^ NKT and myeloid suppressive cells (60, 61), and tumor-derived CCL17 that in turn recruited Tregs already described to have a pro-metastatic role in breast cancer (28).

The function of TSLP in favoring development of primary breast cancer and lung metastasis was subsequently confirmed in the same 4T1 transplantable model where cancer cells were grown in TSLPR KO mice (31). Lack of functional TSLPR mitigated Th2 polarization as well as the establishment and growth of primary breast tumors and lung metastases. Unexpectedly, in the same model brain metastases were found enhanced, suggesting a protective role for TSLP in this site.

In contrast with the results discussed above, Demehri et al. (30) found a tumor-suppressive role for TSLP in murine models of breast carcinogenesis. In order to determine the impact of systemic TSLP on the early stages of breast cancer development, the authors used two murine models. In one model they crossed the MMTV-polyoma middle T (PyMt^tg^) breast cancer-prone with the K14-TSLPtg mice (K14-TSLP^tg^PYMt^tg^), whereas in the other model WT mice were topically treated with calcipotriol, which is known to induce TSLP expression in mouse keratinocytes (62). In both experimental settings breast cancer cells were exposed to high levels of circulating TSLP, were arrested at an early adenoma-like stage, and were prevented from advancing to late carcinoma and metastases. In both models CD4^+^ Th2 cells were shown to mediate the tumor-suppressive effects of TSLP.

A further level of discussion on the pro- vs. anti-tumor role for TSLP in breast cancer was shared by Soumelis and collaborators (63), who did not find TSLP expression in the majority of human tumor samples examined as well as TSLPR expression in tumor infiltrating immune or stromal cells, suggesting lack of TSLP-TSLPR signaling in breast cancer.

### Skin Cancer

Conflicting results were also reported in skin cancer. Demehri et al. (39) reported a tumor-suppressive role for TSLP in skin carcinogenesis by using mice with clonal loss of Notch signaling in their skin. In this model, high levels of TSLP released by barrier-defective skin caused severe inflammation that resulted in gradual elimination of Notch-deficient epidermal clones and resistance to skin carcinogenesis. Overexpression of TSLP in WT skin by chemical induction with calcipotriol also caused resistance to tumorigenesis. As in the breast cancer models reported above, CD4^+^ Th2 cells mediated the tumor-suppressive effect of TSLP in these models of skin carcinogenesis.

In contrast, Takahashi et al. (42) reported that cutaneous T cell lymphoma (CTCL) lesions in advanced stages exhibited a Th2-dominant phenotype. *In vitro* CTCL cell lines and peripheral blood mononuclear cells from Sezary syndrome patients showed increased IL-4 and IL-13 expression in response to TSLP, through the activation of STAT5.

### Gastric Cancer

In gastric cancer patients TSLP expression in the tumor correlated with worse prognosis, and high serum concentration of TSLP was identified as an independent prognostic factor of reduced survival (46). A previous study from the same group (45) had shown that *Helicobacter pylori* infection induced gastric epithelial cells to secrete inflammatory cytokines, among which are TSLP. In addition, *in vitro* DCs conditioned by the supernatant of Helicobacter-infected epithelial cells triggered differentiation of T cells with a mixed Th1/Th2 profile, and TSLP was found to be responsible for the Th2 cytokine production.

### Oropharyngeal Squamous Cell Carcinoma

Finally, analyses of surgical specimens of oropharyngeal squamous cell carcinoma indicated that high IFN-γ and low IL-4, low TSLP, and low TGFβ expression was associated with better prognosis in oropharyngeal squamous cell carcinoma patients (48).

Collectively, in the majority of studies TSLP and Th2 inflammation exerted pro-tumor activity. Conflicting results were reported in pancreatic, breast, and skin cancers.

## Th2-Independent Mechanisms of TSLP in Cancer

In the majority of models Th2-independent mechanisms of TSLP in cancer rely on direct TSLP-TSLPR signaling in TSLPR-expressing tumor cells involving apoptotic pathways, tumor cell proliferation, signal transduction, and activation of remodeling and proangiogenic gene signatures (Table 1).

Th2-independent mechanisms of TSLP in cancer have been reported in breast, lung, cervical, skin, and blood cancers, with pro- and anti-tumor effects, as detailed below.

### Breast Cancer

In breast cancer, three studies (32–34) demonstrated a tumor-promoting role for TSLP. In one study (32), TSLP produced by myeloid cells after activation with tumor cell-derived IL-1α activated anti-apoptotic pathways in TSLPR-expressing tumor cells, through Bcl-2. Experiments in TSLP KO mice then showed that TSLP signaling was required for metastatic disease progression to the lung. In another study (33), tumor cell-derived TSLP induced invasive and angiogenic gene expression profiles in alveolar macrophages. Depletion of alveolar macrophages but not macrophages from the circulation impacted lung lesion growth. A role for TSLP in driving lung metastases was also recently reported in Ragonnaud et al. (34), where tumor cell-derived TSLP induced pre-B cell emigration from the bone marrow through CXCR4 and α4β1 downregulation and promoted their survival and expansion. These pre-B cells were induced by tumor cells to differentiate into regulatory B cells that in turn downmodulated anti-tumor immunity and promoted lung metastases.

### Lung Cancer

A tumor-promoting function for TSLP was described in lung cancer (35), where TSLP expression in the tumor tissue was higher compared to the normal counterpart. *In vitro* experiments showed STAT-1,−3, and−5 phosphorylation in TSLP-DCs that favored recruitment and differentiation of Tregs, possibly through CCL22 and TGFβ secretion, respectively, and in lung cancer patients the prevalence of Tregs correlated with TSLP expression in the tumor.

### Cervical Cancer

Several studies reported a pro-tumor role for TSLP in cervical cancer (36–38). Tumor cells under hypoxia expressed TSLP, and TSLPR was expressed in both tumor cells and vascular endothelial cells. TSLP caused the release of CCL17 by tumor cells with recruitment of eosinophils that in turn induced proliferation and restricted tumor cell apoptosis through up-regulation of Ki-67 and Bcl-2, respectively (36) and of proangiogenic factors (37). TSLP also promoted proliferation and invasion of cervical cancer cells by downregulating microRNA-132 (38).

### Gastric and Ovarian Cancer, and B Cell Precursor-Acute Lymphoblastic Leukemia

A pro-tumor activity for TSLP was described in gastric (44) and ovarian (47) cancer patients where TSLP overexpression in tumor compared to normal tissue correlated with LN metastases (44), and TSLP expression was identified as an independent predictive factor of reduced survival (47). In addition, Vetter and collaborators (49) showed that in a fraction (about 20%) of patients with B cell precursor-acute lymphoblastic leukemia tumor cells expressed the TSLPR, and *in vitro* stimulation of leukemic cells with TSLP enhanced their proliferation and induced activation of STAT-5 signaling.

### Skin Cancer

Conflicting results were instead obtained in skin cancer, where TSLP production by keratinocytes was associated with both pro-tumor and anti-tumor activity. In CTCL, fibroblast-derived periostin mediated TSLP production by keratinocytes that in turn directly stimulated *in vitro* tumor cell growth in TSLPR-expressing tumor cells, and *in vivo* TSLP inhibition reduced tumor formation in EL-4 and MBL-2 cell mouse models (42). A Th2-dependent tumor-promoting role for TSLP in CTCL was also described (see above). On the contrary, in another study Di Piazza et al. (40), using several transgenic and knockout mouse models, demonstrated that TSLP prevented skin carcinogenesis. This effect was mediated mainly by CD8^+^ T cells, possibly because TSLP-TSLPR signaling increased their survival/proliferation. In addition, ablation of the TSLP-TSLPR signaling induced recruitment and/or development of CD11b^+^Gr1^+^ cells that was dependent on epithelial-specific Wnt/β-catenin signaling. These cells directly promoted tumor growth by increased provision of Wnt ligands and not indirectly by acting on T cells. In partial agreement with the report of Di Piazza et al. (40), Cipolat et al. (41) showed that barrier proteins KO (EPI-/-) mice are highly resistant to developing tumors when treated with DMBA and TPA. TPA induced an exaggerated atopic response, immune infiltration, and elevated levels of circulating TSLP. This could be normalized by blocking TSLP or NKG2D but not CD4^+^ T cells. However, it is difficult to explain why mice with lesions > 2 mm had higher levels of TSLP compared with those with lesions < to 2 mm.

### Colorectal Cancer

Finally, an anti-tumor role for TSLP was reported in colorectal cancer (43), where its expression in the tumor was significantly lower than in surrounding tissues, and negatively correlated with clinical staging in colorectal cancer patients. At difference with the anti-apoptotic function reported (2), in this model TSLP enhanced *in vitro* tumor cell apoptosis through caspase-3,−8, and−9 activation, and TSLP administration in xenograft models reduced tumor growth.

Collectively, in the majority of studies through Th2-independent mechanisms, TSLP exerted pro-tumor activity (i.e., breast, lung, cervical, and blood cancers). Conflicting results were reported in skin cancer.

## Conclusion

In the past decade a role for TSLP has been clearly identified in several cancers with somewhat conflicting results, depending on the tumor but even within the same tumor type. In human studies TSLP expression was always associated with a pro-tumor function with the exception of colorectal cancer, whereas an anti-tumor function was found in those mouse models (i.e., pancreatic, breast, and skin cancers), in which high levels of systemic TSLP were reached (Table 1). These data possibly suggest that, independently of the tumor type, also the local vs. the systemic expression of TSLP highly affects its final functional outcomes.

In the majority of studies TSLP-dependent Th2 inflammation was associated with tumor-promoting functions; however, in mouse models of breast and skin carcinogenesis, Th2 cell polarization was associated with tumor-suppressive functions. Possible explanations for these discrepancies can be envisaged. In breast (30) and skin carcinogenesis (39), where transgenic mice express TSLP in their skin keratinocytes, high levels of systemic TSLP were also present (see above), suggesting a possible generalized skew in Th2-type immune responses. Indeed, in these models Th2 cell responses are the only possibly induced tumor-elicited immune responses. Another possible and interesting explanation might be related to different phases of disease development (i.e., early vs. more advanced stages). It has been reported (64) that IL-13 derived from intraepithelial lymphocytes regulates tissue homeostasis during skin injuries and protects against skin carcinogenesis. It is tempting to speculate that, especially at barrier sites, Th2 cell responses might be relevant in early stages carcinogenesis when tissue repair is ongoing. However, when tumors are established, Th2 cells/cytokines become not only insufficient compared to Th1 cells/cytokines as anti-tumor effectors but also promote a chronic tissue repair program, which facilitates the activation of a pro-angiogenic and pro-metastatic tumor microenvironment.

Recently, asthma exacerbations were prevented by an anti-TSLP monoclonal antibody (65), making this therapy also available in tumor types, in which a proven tumor-promoting role of TSLP has been established. In addition, preclinical evidence also suggested the possibility to manipulate the TSLP secretion by modulation of its production. Indeed, IL-1 was shown to be a key factor for activation of TSLP secretion in both pancreatic (22) and breast cancer (21), where the use of the IL-1R antagonist anakinra reduced TSLP availability *in vitro* and in vivo. On the opposite side, treatment with calcipotriol, which increases TSLP levels, in combination with 5-fluorouracil was superior to combination with Vaseline in reducing actinic keratosis lesions (66).

Collectively, whereas a role for TSLP in cancer is firmly established, manipulation of its expression for therapeutic purposes will need further definition of its pro-tumor vs. anti-tumor function in the different tumor types.

## Author Contributions

All authors listed have made a substantial, direct and intellectual contribution to the work, and approved it for publication.

## Conflict of Interest

The authors declare that the research was conducted in the absence of any commercial or financial relationships that could be construed as a potential conflict of interest.

## Abbreviations

CAF: cancer associated fibroblasts
CTCL: cutaneous T cell lymphoma
DC: dendritic cell
LN: lymph node
TDLNs: tumor-draining LNs
Tregs: regulatory T cells
TSLP: thymic stromal lymphopoietin
TSLPR: TSLP receptor
WT: wild-type.
